# Associations between neighborhood built, social, or toxicant conditions and child externalizing behaviors in the Detroit metro area: a cross-sectional study of the neighborhood ‘exposome’

**DOI:** 10.1186/s12889-022-13442-z

**Published:** 2022-05-28

**Authors:** Amber L. Pearson, Elizabeth A. Shewark, S. Alexandra Burt

**Affiliations:** 1 Department of Geography, Environment and Spatial Sciences, Michigan State University, East Lansing, USA; 2 Department of Psychology, Michigan State University, East Lansing, USA

**Keywords:** Mental health, Greenspace, Pollution, Post-industrial, Exposome

## Abstract

**Background:**

The specific ‘active ingredients’ through which neighborhood disadvantage increases risk for child psychopathology remains unclear, in large part because research to date has nearly always focused on poverty to the exclusion of other neighborhood domains. The objective of this study was to evaluate whether currently assessed neighborhood built, social, or toxicant conditions were associated with child externalizing psychopathology outcomes separately, and in a combined model, using data from the Detroit-metro county area.

**Methods:**

We conducted principal components analyses for built, social, or toxicant conditions. Next, we fitted separate multiple regression models for each of the child externalizing psychopathology measures (oppositional defiant and conduct problems) as a function of built, social, or toxicant components.

**Results:**

We found that built features (more non-profits, churches, and alcohol outlets, and less agriculture and vacant properties) were associated with conduct problems, while toxicant conditions (high percent industrial, toxins released and number of pre-1978 structures) were associated with oppositional defiance problems. There was no significant association between greenspace or social conditions and child externalizing outcomes. When examined simultaneously, only the significant independent association between built conditions and conduct problems remained.

**Conclusions:**

Built, social, and toxicant neighborhood conditions are not interchangeable aspects of a given neighborhood. What’s more, built features are uniquely associated with child externalizing outcomes independently of other neighborhood characteristics. Future research should consider how changes in the built conditions of the neighborhood (e.g., development, decay) serve to shape child externalizing behaviors, with a focus on identifying potentially actionable elements.

**Supplementary Information:**

The online version contains supplementary material available at 10.1186/s12889-022-13442-z.

## Background

Neighborhood disadvantage is a potent predictor of maladaptive behavioral and emotional outcomes in children across development [1–13], including externalizing (e.g., antisocial behavior, oppositional defiant) symptoms [11, 14–19]. Links between neighborhood disadvantage and externalizing outcomes emerge early in life and importantly, increase over time [20]. This is likely due to increases in agency starting in middle childhood and adolescence [21, 22]. As individuals begin to move through their environments independently, they are exposed more directly to their neighborhood environments. For instance, when comparing antisocial behavior in children living in deprived versus affluent neighborhoods at age 5, the Cohen’s *d* was 0.38 [20]. By age 12, the Cohen’s *d* in those same youth was 0.51 [20]. This amplification of the links between neighborhood disadvantage and youth externalizing outcomes over time has very important downstream effects, as youth with externalizing outcomes are at high risk for academic delay/dropout, substance abuse, under/unemployment, and incarceration in adulthood [23–25]. What’s more, externalizing behaviors in children are shown to predict not only disruptive disorders in adulthood, but also anxiety, mood, and substance use disorders [26] and work incapacity [27].

To date, however, the specific ‘active ingredients’ through which disadvantage increases risk for child externalizing outcomes remain unclear, in large part because relevant child developmental research to date has nearly always assessed disadvantage at the child- or family-specific level, despite recent advances in geospatial data availability and analytical techniques for quantifying neighborhood context. When studies do include neighborhood disadvantage, the vast majority of studies conceptualize disadvantage almost exclusively in terms of neighborhood poverty, neglecting other important dimensions of neighborhood which may relate to child outcomes. Indeed, a handful of researchers have extended this work to explore the effects of other neighborhood social conditions, beyond poverty, on child health and development, including collective efficacy [28] (defined as the shared belief to organize and carry out collective action [29] and neighbor perceptions of disadvantage [30]. In our research, for example, we found that environmental influences on children’s rule-breaking antisocial behavior were several-fold larger when they resided near neighbors with high levels of rule-breaking themselves, whereas genetic influences were more influential in the absence of neighbors with high levels of rule-breaking [31]. Moreover, this etiologic moderation appeared to be driven by geographic proximity to neighbors. Such findings strongly suggest that neighborhood social conditions beyond census poverty measures are important for the etiology of child externalizing behaviors.

Despite the extension of this research into other domains of the neighborhood social context, studies examining child externalizing outcomes to date have largely failed to consider the effects of built environments (i.e., human-made features such as alcohol outlets) within disadvantaged communities. This is an important gap in the literature, since the few studies examining the built environment have suggested that child externalizing outcomes may be affected by signs of disorder (e.g., graffiti, broken windows, vacant lots) **[**32**–**34**]**, noisy roads and airports [35], and the absence of greenspaces [36–39].

Extant studies have also failed to consider the extent to which the toxicant environment (i.e., pollutants in the soil, air and water) directly affects and/or mediates the association between neighborhood social disadvantage and child externalizing outcomes, although there is some evidence this may be the case. A review of the evidence regarding air pollutants (e.g., polycyclic aromatic hydrocarbons, PM_2.5_, and nitrogen oxides) concluded there are clear, negative impacts on the neuropsychological development of children [40]. Another such exposure is chronic, early-life exposure to lead, which demonstrates robust links to several forms of maladaptive behavioral and emotional outcomes (e.g., [41, 42]) and strongly covaries with neighborhood disadvantage [42]. What’s more, pollutants and built environment risks also tend to follow historical patterns of socioeconomic disadvantage with less neighborhood greenspace [43], higher density of tobacco retailers [44], alcohol outlets [45] and vacancy [46], and poorer food access [47]. Still, existing studies tend to evaluate singular measures of the neighborhood in relation to child health, rather than evaluating a constellation of neighborhood factors in tandem. Indeed, only a few studies have jointly examined social, built, and toxicant neighborhood domains, and even fewer have examined multiple possible active ingredients within those domains. Of the studies examining multiple aspects of the neighborhood, none to our knowledge focused on child externalizing behaviors. Rather, studies have found associations between multiple aspects of neighborhoods and child weight-related behaviors [48], incarceration and teenage parenthood [49], and adiposity [50].

In sum, several independent lines of research point to important effects of the social, built, and toxicant neighborhood environments on child externalizing outcomes. Critically, however, these neighborhood domains are nearly always evaluated separately. This is a huge gap in the literature, as we would in fact expect neighborhood built, social, and toxicant environments to be associated withchild externalizing outcomes *in tandem*. In an effort to evaluate the totality, or as near as feasible, of environmental exposures that individuals experience, researchers have begun to argue for measurement of the ‘exposome’ [51]. Measurement of the exposome often entails multiple sources and types of data, including sensors, geographic information systems (GIS), remotely sensed imagery, and conventional surveys. To date, however, we know of no study examining neighborhood exposome effects on child externalizing outcomes. It is thus unclear whether and how the various elements of the neighborhood exposome might jointly or synergistically influence child health outcomes.

Using geospatial data compiled from various sources by all authors and child mental health data compiled by the senior author (SAB), the objective of this study was to evaluate whether neighborhood built, social, or toxicant conditions were associated with child externalizing outcomes. Since consideration of multiple domains of the neighborhood is a novel endeavor, we did not have explicit hypotheses specifying how each domain of neighborhood conditions might differentially associate with externalizing outcomes. The selection of neighborhood features compiled here was a balance between existing evidence and fine spatial and temporal availability of data. We conducted this study in the Detroit-metro county area (Wayne, Macomb, Oakland, Livingston, and Washtenaw counties), which is diverse in terms of economic context, built features, industrial histories, neighborhood disadvantage, and ethnic composition.

## Methods

### Human subjects approval

The Michigan State University institutional review board approved this study (STUDY00004447).

### Participants

Participants were drawn from the Twin Study of Behavioral and Emotional Development in Children (TBED-C), a study within the population-based Michigan State University Twin Registry (MSUTR) [52]. To be eligible for participation in the TBED-C, neither twin could have a cognitive or physical condition as assessed via parental screen (e.g., a significant developmental delay) that would preclude completion of the assessment. Children provided informed assent, while parents provided informed consent for themselves and their children. The TBED-C includes both a population-based sample (*n* = 528 families) and an independent ‘at-risk’ sample (*n* = 502 families). Additional inclusion criteria for the ‘at-risk’ sample specified that participating twin families lived in neighborhoods with Census-level poverty above the 2008 mean of 10.5%. This recruitment strategy yielded overall response rate of 57% for the at-risk sample and 63% for the population-based sample [52]. For the current study, only those TBED-C participants residing in the five-county study area (*n* = 720 children in 360 families; 35.0% of families participating in the TBED-C) were included. Age, sex, and ethnicity information on the children is presented in Table 2.

### Geospatial neighborhood data

For the five counties in our study, we compiled data related to the built, social, and toxicant conditions in neighborhoods (Table 1), at a time point as close to the intake assessment conducted for the TBED-C [52] (2008-2014) and ideally at the middle point in this time period (2011). We first geocoded child addresses and conducted spatial accuracy checks on 57 random geocoded addresses. Using Google Earth as the gold standard, we found that geocoded locations were 2.08 to 6250.49 m different from Google Earth locations (*M* = 370.83, *SD* = 1232.01). Based on our previous research in Michigan [30], we defined ‘neighborhood’ as a 5 km extent from the home location of each child (as Euclidean distance). All spatial techniques were conducted using ArcGIS v10.6 (ESRI, Redlands, CA). Spatial data, when possible, were compiled for the midpoint (2011) in the participant recruitment time period (2008-2014). When not possible, the nearest time period was selected.

**Table 1:** Sources of neighborhood data compiled in this study, within the five Detroit Metro counties

Construct	Measure	Instrument or source of data	Time period
Built conditions	Alcohol outlets	Liquor Control Board licenses and from the USA reference database (https://lib.msu.edu/about/data/referenceusahistorical/), *n* = 14,916	Business primary revenue source (2011)
	Vacant properties	Tax parcel data for each jurisdiction, coded as vacant, *n* = 113,028	2011
	Agricultural land use	Tax parcel data for each jurisdiction, coded for land use, *n* = 321,405	2011
	Greenspaces	ESRI, *n* = 870	2018
	Churches	Urban Institute National Center for Charitable Statistics Data Archives (https://nccs-data.urban.org/data.php?ds=core), *n* = 264	2011
	Non-profit organizations	Urban Institute National Center for Charitable Statistics Data Archives (https://nccs-data.urban.org/data.php?ds=core), *n* = 1657	2011
Social conditions	Perceived social process	Questionnaires administered to neighbors via Michigan Twins Study, mean number of neighbors per family = 16.04 (SD = 11.48, range of 1 to 45), social cohesion and social control	2008-2014
	Poverty	Area Deprivation Index, University of Wisconsin, *n* = 3773	2015
		ACS 5-year estimates	2008-2012
Toxicant conditions	Quantities of industrial toxicants	ToxMap EPA Tri-facilities, combined toxicant releases (https://toxmap.nlm.nih.gov/toxmap/download.html), *n* = 804	Cumulative values 1988 to 2016
	Industrial land use	Tax parcel data for each jurisdiction, coded for land use, *n* = 321,405	2011
	Lead from housing	Structures built prior to 1978, *n* = 922,165	Compiled in 2020, includes age of all structures

### Alcohol outlets

We collected alcohol outlet data through the USA reference database (ReferenceUSA, 2005-2018). The reference database records all primary and secondary revenue sources for businesses in the United States since 1997. For these data, we selected all businesses in Michigan that listed alcohol as a primary revenue source (this includes stores that sell packaged alcohol and places of on-site consumption). We call these alcohol outlets and extracted all outlets for 2011. We then summed the number of outlets within each child’s buffer and calculated the distance (in meters) from each child’s home to the nearest alcohol outlet.

### Vacant properties and land use

Using the Southeast Michigan Council of Governments open data portal, we compiled land use data for 2011, which includes vacant properties. We calculated the percent land area covered by vacant properties within each child’s neighborhood extent. We also calculated the percent of industrial and agricultural land uses within each child’s buffer.

### Greenspaces

We compiled all parks, gardens, and forests within the United States at national, state, county, regional, and local levels from ESRI in 2018. We then calculated the percent land area covered by greenspaces within each child’s buffer and calculated the distance from the child’s home (in meters) to the nearest greenspace.

### Churches and non-profit organizations

We collected data from the NCCS Data Archive (Urban Institute, National Center for Charitable Statistics, 2005-2014) which denotes places of worship and other non-profit organizations based on tax documents. For each child, we calculated the number of churches/places of worship and other non-profits within the neighborhood and the distance to the nearest church/non-profit from the home location (in meters).

### Neighborhood social processes

Neighborhood cohesion (30 Items assessing perceptions of support and help among neighbors; *α* = 0.95) and informal social control (29 items assessed perceptions that community residents will maintain social order; *α* = 0.91) were assessed using the Neighborhood Matters questionnaire [53]. For these scales, higher values indicate higher cohesion and social control. The questionnaire was completed by participants and their neighbors. Neighbors were recruited as follows: after the participation of a given family in the study, we sent mailings to 10 randomly-chosen addresses in that family’s Census tract, inviting one adult resident per household to complete a survey. When a particular randomly-chosen address was no longer inhabited (i.e., the letter was returned as undeliverable), one attempt was made to find a replacement address. This approach resulted in a sample of 1880 neighbors (63.2% women; 80.6% White, 11.6% Black, 7.8% other ethnic group memberships; average age of 52.6 with a range of 18-95 years). The response rate was 70%, of which 70% agreed to participate (for a final participation rate of 49%). Of these, 411 neighbors resided in the Detroit area, and were thus eligible for the current study. Children were assigned the average values of the nearest five neighbors within the 5 km neighborhood buffer.

### Poverty

We used the 2015 Area Deprivation Index (ADI) which is a score composed from 17 Census variables obtained between 2009 and 2014, compiled at the Census block group level [54]. We used the Michigan-specific rank score, whereby higher values indicate higher levels of deprivation. We assigned children the value of the polygon in which their home location was located. We also used neighborhood poverty data from the American Community Survey 5-year estimates (2008-2012) of the percent of households with children living below poverty level.

### Industrial toxicants

Toxicant data were collected via the Toxmap from NLM of National Institutes of Health. We compiled toxicant releases cumulative from 1988 to 2016. For each child, we summed the total lead released from all point sources located within the neighborhood and the distance to the nearest site that released lead (in meters).

### Lead from housing

We compiled tax parcel data, which includes the year built for each structure. For each child, we summed the number of structures that were built prior to 1978 within their neighborhood extent. Lead paint, commonly used on housing exteriors, was banned in 1978, and thus the number of buildings built prior to that time can be considered an index of lead paint dust exposure in the area.

### Child mental health and covariate data

We captured informant-reports of each child’s emotional and behavioral outcomes using up to four reporters for each child: mother, child, teacher, and father. Mothers and fathers completed the Child Behavior Checklist (CBCL; *α* = 0.76 to 0.79) [55], which is a 113 item questionnaire assessing children’s competencies and behavioral problems. Teachers completed the Teacher Report Form (TRF; *α* = 0.85 to 0.86) [55]. Comparable to the CBCL, the TRF is 113 item questionnaire that assesses children’s competencies and behavioral problems. Last, children completed the Achenbach Semistructured Clinical Interview for Children and Adolescents (SCICA) [56]. The interview is a standardized assessment of children’s behaviors and is designed to be compatible with the CBCL and the TRF. Roughly 10% of SCICA interviews were videotaped to obtain inter-rater reliability, and the average intraclass correlation across raters was .88. Across these measures, we focused on DSM-oriented oppositional defiant problems and conduct problems scales.

We averaged informant reports for each scale to create a multi-informant composite of each outcome. The decision to average informant reports comes from prior work showing that each informant is providing incrementally valid information regarding the child’s behavior. Prior meta-analyses of informant effects [57] have shown that various informant-reports of a given child’s psychopathology tend to evidence only small-to-moderate associations, likely as a function of the different informants’ exposures to different slices of the child’s behavior [58]. For example, parents of school-aged children typically observe their children in less structured home settings and are privy to only some of what happens during the school day, whereas teachers observe children in a more rigid classroom setting and have a clearer sense of developmental norms for children that age. When they can be reliably and validly assessed, the children are also very useful informants, in that they are explicitly motivated to conceal antisocial behaviors from adults and thus have unique knowledge of antisocial acts for which they were not caught. In these data, the various informant-reports were only moderately intercorrelated in these data (*r*s among reports of youth MBEO ranged from .19 to .57; all *p* < .01; see Supplementary Tables S7-S8), results that are very much in keeping with prior meta-analytic data [57], and with the interpretation that different informants are exposed to different slices of the child’s behavior. However, to further assess the utility of this approach in these data, we evaluated the underlying structure of informant reports with confirmatory factor analyses. Results indicated that a single factor for both oppositional defiance and conduct problems, respectively (see Supplementary Tables S31-S32). Given these considerations, we have adopted a combined informant approach whenever possible, which is thought to allow for a more complete assessment of child symptomatology than would the use of any one informant alone [57].

Demographic data on child age, sex, and ethnicity was captured at recruitment via survey. Previous MSUTR analyses have found some significant effects for these covariates and thus we controlled for them herein [59]. Ethnicity was recoded into a binary variable to assist with interpretation (White versus non-White). Sex was coded such that 1 indicated those that identified as male and − 1 indicated those that identified as female. We also included population density for the Census block in which the child resides as an independent variable for account for potential rural/urban contexts.

### Statistical analyses

First, we calculated descriptive statistics for participants and their neighborhoods, stratified by high/low neighborhood poverty status, using the median of 11.30 as the threshold. The median was used due to considerable positive skew (1.78) in the data. Next, we calculated Pearson’s r and *p*-values for correlations between neighborhood variables. To address our study objective, we first conducted principal components analyses (PCA) for each of the three domains: built, social and toxicant neighborhood conditions. Indicators evidencing significant skew were log transformed prior to conducting the PCA (see Supplementary Materials Tables S1-S4 for descriptive statistics). Only components with eigen values > 1 were extracted (see Supplementary Tables S24-S27). For the built conditions, we generated a single component which included non-profits churches, alcohol outlets, agricultural land use, and vacant properties. Greenspace was considered as a separate independent variable, as it formed its own component in our PCA. For social conditions, we generated a single component score which included state-level area socioeconomic deprivation, neighborhood poverty, social cohesion, and informal social control. Last, for the toxicant conditions, we created a single component that included the total toxicants released from all EPA-monitored Tri-facilities from 1988 to 2016, industrial areas, and number of pre-1978 structures. PCA was conducted using SPSS 27 software (IBM Corp., 2020).

Next, we fitted separate multiple regression models for each of the child externalizing measures (oppositional defiant problems and conduct problems) as a function of built, social, or toxicant conditions, evaluated individually. To account for the non-independence of twins within the same family, we used clustering with robust standard errors. Mother (1.4%) and child (1.1%) informants had minimal amounts of missing data. However, father (23.6%) and teacher (25%) informants had a larger amount of missing data. To handle missing data, we used MLR estimation as is recommended with cluster-robust standard errors [60]. We included ethnicity (white vs non-white), sex, and age as covariates in the models. Greenspace and conduct problems had considerable skew and were log transformed prior to analysis. To account for multiple testing across two forms of externalizing, we Bonferroni-corrected our significance level (adjusted *p*-value is .025). Our final analyses assessed the unique contributions of the built, social, and toxicant conditions by fitting a single multiple regression model with all independent variables separately for each child mental health outcome. Greenspace was not included in these analyses because it was not a significant predictor of child externalizing in the first set of analyses. As sensitivity analyses, we evaluated neighborhood effects using a 1 km buffer size, but found similar results (see Supplementary Materials Tables S5-S6; S15-S23; and S28-S30). We also evaluated potential interactions between independent variables but did not detect significant associations (see Supplementary Materials Table S14). Mplus 8 was used for all regression modelling (Muthén & Muthén, 1998-2019).

## Results

Differences by neighborhood poverty were small for age, neighborhood vacant area, and conduct problems (Table 2). Higher poverty neighborhoods had higher percentage African American children, more non-profits, churches and alcohol outlets, less greenspace and agricultural land and about twice the levels of toxicants and about twenty times the number of old structures. Higher poverty neighborhoods also had less industrial land and children with higher oppositional defiant problems. In examining correlations between specific neighborhood variables, we observed moderate (> 0.5) positive correlations between alcohol outlets, non-profits and churches (Table 3). We also observed a negative, moderate correlation between percent agricultural area and alcohol outlets. At the broader domain level, we observed moderate-to-large correlations at the 5 km level (*r*s were .55 between built and social domains, .63 between social and toxicant domains, and .27 between built and toxicant domains; all *p* < .01, see Supplementary Materials Table S9). When examining smaller areas (1 km), we saw even smaller and sometimes negatively-signed correlations (*r*s were .18 between built and social domains, −.20 between social and toxicant domains, and − .27 between built and toxicant domains; all *p* < .01, see Supplementary Materials Table S9).

**Table 2:** Descriptive statistics for demographic and neighborhood conditions of children in our sample, stratified by high/low poverty status

	High poverty neighborhoods (*n* = 354 children)	Low poverty neighborhoods (*n* = 364 children)	Total (*n* = 720 children)
Demographics			
Age^b,^ mean (sd)	8.2 (1.6)	8.2 (1.4)	8.20 (1.49)
Ethnicity, %			
White	61.0	86.3	73.9
African American	28.8	5.5	16.9
Asian	1.1	1.6	1.4
Other	9.1	6.5	7.8
Female, %	48.9	52.5	50.8
Built conditions			
Non-profits, mean (sd)	35.8 (30.3)	25.8 (21.4)	30.7 (26.6)
Churches, mean (sd)	5.4 (3.9)	4.3 (4.2)	4.9 (4.1)
Alcohol outlets, mean (sd)	282.2 (147.7)	192.4 (130.9)	236.1 (146.6)
Percent area - greenspace, mean (sd)	4.2 (3.9)	4.7 (4.7)	4.4 (4.3)
Percent area - agricultural land use, mean (sd)	4.1 (11.5)	7.9 (15.8)	6.1 (13.9)
Percent area - vacant properties, mean (sd)	9.0 (4.8)	8.7 (4.8)	8.8 (4.8)
Social conditions			
Area deprivation index^a^, mean (sd)	6.1 (2.8)	2.9 (2.3)	4.5 (3.0)
Neighborhood poverty^c^, mean (sd)	28.4 (16.7)	5.6 (3.2)	16.8 (16.5)
Social cohesion^d^, mean (sd)	102.8 (7.4)	105.5 (10.1)	104.0 (8.8)
Social control^d^, mean (sd)	24.2 (2.3)	25.5 (1.8)	24.8 (2.2)
Toxicant conditions			
Total toxicants 1988-2016 in millions, mean (sd)	8.1 (12.8)	4.1 (9.1)	6.1 (11.2)
Structures built before 1978 in 1000s, mean (sd)	42.1 (3.1)	2.1 (23.1)	31.5 (29.4)
Percent area - industrial land use, mean (sd)	15.7 (5.2)	17.9 (3.2)	4.5 (3.7)
Health measures			
Oppositional defiant problems^e^, mean (sd)	3.4 (1.8)	2.9 (1.6)	3.1 (1.7)
Conduct problems^e^, mean (sd)	1.5 (1.9)	1.4 (1.7)	1.5 (1.8)

^a^Range 1-10, where 10 indicates high deprivation

^b^Range 6 years to 11 years

^c^This is the percent of households with children living below poverty

^d^Higher values indicate more cohesion and control, range 58 to 142 and 16.3 to 29, respectively

^e^Higher values indicate child mental health problems, ranges (oppositional 0 to 10.3), (conduct 0 to 14)

**Table 3:** Correlations between neighborhood variables

	1	2	3	4	5	6	7	8	9
1. Number non-profits	–	.25^**^	**0.05**	.44^**^	.14^**^	**0.03**	.13^**^	−0.18**	−.20^**^
2. Number churches	.55^**^	–	**− 0.01**	.25^**^	.18^**^	**0.05**	**−0.03**	−.14^**^	−.09^*^
3. Sum toxicants	.08^*^	−0.01	–	**0.07**	**0.04**	**− 0.02**	.25^**^	**0.04**	**−.00**
4. Number alcohol outlets	.72^**^	.65^**^	0.04	–	.55^**^	**−0.04**	.15^**^	−.33^**^	−.19^**^
5. Number pre1978 structures	.33^**^	.44^**^	−0.04	.81^**^	–	**0.02**	.11^**^	−.33^**^	−.14^**^
6. Percent green space area	−.20^**^	−.20^**^	−0.05	−.23^**^	−.17^**^	–	.22^**^	−.14^**^	.13^**^
7. Percent industrial area	.27^**^	.13^**^	.49^**^	.33^**^	.20^**^	−.12^**^	–	−.11^**^	.14^**^
8. Percent agricultural area	−.41^**^	−.40^**^	−.13^**^	−.59^**^	−.42^**^	−.09^*^	−.36^**^	–	.14^**^
9. Percent vacant lots	−.31^**^	−.25^**^	.27^**^	−.33^**^	−.25^**^	.12^**^	.09^*^	0.01	–

Correlations among raw variables. *p < .05, **p < .01; 5 km below the diagonal and 1 km above the diagonal (in bold). Poverty and area deprivation index not included, as these were measured at the Census tract and block group levels, respectively

In exploring whether neighborhood built, social, or toxicant conditions were individually associated with child externalizing behaviors, we found that built neighborhood conditions (more non-profits, churches, and alcohol outlets, and less agriculture and vacant properties) were significantly associated with conduct problems (Table 4) and the association with oppositional defiance problems was approaching statistical significance. In fact, built conditions exhibited moderate to large associations with both outcomes, when compared to other independent variables (ethnicity, sex, age, and population density). There was no significant association between greenspace (Table 5) or social conditions (high disadvantage and poverty, low cohesion and social control; Table 6) and child externalizing behaviors. However, for toxicant conditions (high percent industrial, toxins released and number of pre-1978 structures), we observed a large, significant, and positive association with oppositional defiance problems (Table 7). However, in the combined model, we only found a significant independent association between built conditions and conduct problems, when accounting for demographic characteristics and other neighborhood conditions (Table 8). The effect size was about half that of child sex.

**Table 4:** Child health measures and neighborhood built conditions

	Oppositional Defiance Problems					Conduct problems				
	B	SE B	β	95% CI	*P*	B	SE B	*β*	95% CI	*p*
Ethnicity	0.23	0.09	0.12	(.03, .21)	0.011*	0.07	0.03	0.10	(.01, .19)	0.032
Sex	0.25	0.07	0.15	(.07, .23)	< 0.001*	0.15	0.02	0.26	(.18, .34)	< 0.001*
Age	0.07	0.05	0.06	(−.03, .14)	0.178	−0.04	0.02	−0.11	(−.19, −.03)	0.010*
Population density	0.14	0.10	0.07	(−.03, .16)	0.182	−0.02	0.04	−0.03	(−.13, .07)	0.543
Built conditions	0.18	0.08	0.11	(.01, .20)	0.029	0.08	0.03	0.15	(.06, .23)	0.002*
AIC = 12,802.52; BIC = 12,926.16						AIC =11,235.49; BIC = 11,359.12				

*With Bonferroni correction, the adjusted *p*-value for significance is 0.025

**Table 5:** Child health measures and neighborhood greenspace

	Oppositional Defiance Problems					Conduct problems				
	B	SE B	β	95% CI	*P*	B	SE B	*β*	95% CI	*p*
Ethnicity	0.25	0.09	0.13	(.04, .22)	0.008*	0.07	0.03	0.11	(.02, .20)	0.019*
Sex	0.25	0.07	0.15	(.07, .23)	< 0.001*	0.15	0.02	0.26	(.18, .34)	< 0.001*
Age	0.07	0.05	0.06	(−.03, .15)	0.168	−0.04	0.02	−0.10	(−.18, −.02)	0.014*
Population density	0.25	0.09	0.12	(.04, .20)	0.006*	0.03	0.03	0.04	(−.05, .13)	0.354
Green space	0.02	0.10	0.01	(−.07, .09)	0.814	0.01	0.03	0.01	(−.07, .08)	0.861
AIC = 12,502.42; BIC = 12,626.06						AIC =10,941.50; BIC = 11,065.14				

*With Bonferroni correction, the adjusted *p*-value for significance is 0.025

**Table 6:** Child health measures and neighborhood social conditions

	Oppositional Defiance Problems					Conduct problems				
	B	SE B	β	95% CI	*p*	B	SE B	*β*	95% CI	*p*
Ethnicity	0.18	0.10	0.09	(−.00, .19)	0.066	0.07	0.04	0.10	(.00, .21)	0.049
Sex	0.25	0.07	0.15	(.07, .22)	< 0.001*	0.15	0.02	0.26	(.18, .34)	< 0.001*
Age	0.07	0.05	0.06	(−.03, .15)	0.165	−0.04	0.02	−0.1	(−.18, −.02)	0.013*
Population density	0.19	0.10	0.09	(.00, .19)	0.048	0.03	0.04	0.04	(−.06, .14)	0.462
Social conditions	0.14	0.10	0.08	(−.03, .19)	0.155	0.01	0.03	0.01	(−.10, .12)	0.796
AIC = 12,401.75; BIC = 12,525.39						AIC =10,843.62; BIC = 10,967.26				

*With Bonferroni correction, the adjusted *p*-value for significance is 0.025

**Table 7:** Child health measures and neighborhood toxicant conditions

	Oppositional Defiance Problems					Conduct problems				
	B	SE B	β	95% CI	*p*	B	SE B	*β*	95% CI	*p*
Ethnicity	0.14	0.09	0.07	(−.02, .17)	0.141	0.05	0.03	0.07	(−.02, .11)	0.176
Sex	0.23	0.07	0.14	(.06, .21)	0.001*	0.14	0.02	0.25	(.10, .19)	< 0.001*
Age	0.06	0.05	0.05	(−.03, .14)	0.226	−0.04	0.02	−0.11	(−.07, −.01)	0.009*
Population density	0.09	0.1	0.04	(−.05, .14)	0.364	−0.01	0.04	0.01	(−.09, .07)	0.804
Toxicant conditions	0.25	0.11	0.15	(.03, .27)	0.019*	0.06	0.03	0.11	(−.00, .13)	0.064
AIC = 12,516.30; BIC = 12,639.94						AIC =10,959.12; BIC = 11,082.76				

*With Bonferroni correction, the adjusted *p*-value for significance is 0.025

**Table 8:** Child health measures and all neighborhood conditions combined (except greenspace)

	Oppositional Defiance Problems					Conduct problems				
	B	SE B	β	95% CI	*p*	B	SE B	β	95% CI	*p*
Ethnicity	0.12	0.10	0.06	(−.03, .16)	0.198	0.05	0.04	0.08	(−.02, .19)	0.121
Sex	0.24	0.07	0.14	(.06, .22)	< 0.001*	0.15	0.02	0.26	(.18, .33)	< 0.001*
Age	0.06	0.05	0.06	(−.03, .14)	0.203	−0.04	0.02	−0.11	(−.18, −.03)	0.009*
Population density	0.04	0.11	0.02	(−.08, .12)	0.734	−0.04	0.04	−0.05	(−.16, .07)	0.395
Built conditions	0.13	0.09	0.08	(−.03, .18)	0.167	0.07	0.03	0.13	(.03, .23)	0.010*
Toxicant conditions	0.16	0.13	0.09	(−.06, .24)	0.233	0.03	0.04	0.06	(−.08, .19)	0.413
Social conditions	0.10	0.11	0.56	(−.07, .18)	0.390	−0.01	0.04	−0.01	(−.13, .11)	0.897
AIC = 15,549.82; BIC = 15,751.30						AIC = 13,987.60; BIC = 14,189.09				

*With Bonferroni correction, the adjusted *p*-value for significance is 0.025

Although they were somewhat peripheral to our core focus on child externalizing disorders, we also conducted supplemental analyses examining the associations of internalizing behaviors (affective and anxiety problems) and ADHD with neighborhood conditions. No significant associations with neighborhood conditions were detected (see Supplementary Materials Tables S10-S13). However, should we further adjust the above Bonferroni correction to account for these disorders as well, the *p*-value for significance would become .01 (versus .025). Although this change would move the association between toxicant conditions and oppositional defiant problem to trend level, the association between built conditions and conduct problems would remain statistically significant.

We also conducted two sets of post-hoc sensitivity analyses. First, we evaluated neighborhood effects using a 1 km buffer size. Results were very similar to those reported above (see Supplementary Materials Tables S5-S6; S15-S23; and S28-S30), suggesting that our results are robust to more than one operationalization of ‘neighborhood’. Second, we evaluated potential interactions between independent variables. None of the interactions were significant (see Supplementary Materials Table S14).

## Discussion

We were able to identify several neighborhood features within the built, social, and toxicant domains that grouped together to form components. These three domains of neighborhood characteristics evidenced moderate-to-large positive correlations at the 5 km level (*r*s ranged from .27 to .63), but smaller and sometimes negatively-signed correlations when examining a smaller area (1 km; *r*s ranged from −.27 to .18). Regardless, such findings are clearly consistent with our contention that various neighborhood conditions are not interchangeable, an observation that appears to be especially relevant in more localized areas. Furthermore, child outcomes could be predicted by two of these domains when analyzed separately. Although some of the associations may relate to urban/rural differences within Michigan, we note that population density was rarely a significant predictor when adjusting for neighborhood conditions. Still, urban settings tend to have higher numbers of amenities (churches and non-profits) and lower levels of agricultural land use. These features also tend to concentrate in less advantaged neighborhoods and were associated with poorer child outcomes. When examining all three domains together, the built environment showed an independent association with conduct problems, even after adjustment for social conditions (including poverty) and toxicant conditions. Interestingly, there were no independent associations between social conditions and the two externalizing outcomes. This is surprising given that most extant research has focused on economic disadvantage, and has found consistent evidence of small-to-moderate associations. We also did not observe statistically significant associations between greenspace and child outcomes.

Taken together, these findings suggest built, social, and toxicant neighborhood conditions are not interchangeable, and that built features influence child externalizing outcomes independently of other neighborhood characteristics. Laboratory and experimental research suggests that, like nearly all species, humans process visual cues in their surrounding environment as either threatening or non-threatening, influencing behavior, sympathetic and parasympathetic nervous activity, and ultimately stress or recovery from stress [61–64]. Since features of the built environment are inherently visual, these features may be particularly relevant to children, who spend the majority of their leisure time near the home [65]. This is an important set of findings, given that much extant research in this area has been solely focused on poverty to the exclusion of the built environment.

We were also surprised to find that greenspace was not associated with our externalizing measures, given prior research [36–39]. One possible explanation for this is that greenspace quantity may be less important than quality in influencing child externalizing per se [66]. This may be particularly important in post-industrial areas, where vacant lots and unmaintained greenspaces may be more prevalent [67, 68]. We did find a statistically significant effect of toxicant conditions on oppositional defiance problems. The lack of a significant association between toxicant conditions and conduct problems was surprising given the evidence showing the influence of lead exposure on conduct problems [69]. These null results may reflect challenges of assessing individual exposure to lead using neighborhood-level measures, indicating that direct exposure measures (e.g., blood tests) may be optimal.

### Strengths and weaknesses

This study examined child externalizing data from a large sample of children oversampled for residence in low-income neighborhoods. The sample includes children living in both major cities (e.g., Detroit) and many rural areas in Michigan. We used a child-specific neighborhood, rather than census-defined neighborhood, which may be more relevant for children’s everyday exposures near the home [65]. Still, there are weaknesses to this study. First, this study is cross-sectional, meaning that no causality can be inferred from these findings. Future longitudinal studies may provide important insights. Second, while significant effort was spent to obtain data on numerous neighborhood features at the finest temporal and spatial scale possible, not all possible neighborhood features could be included. There may indeed be other relevant neighborhood features worth considering in future research. For example, perceived safety or fear of crime [70–73], exposure to violence [74], ethnic heterogeneity or segregation [75], and exposure to racism and discrimination [76] could also be important to consider. Likewise, it may be that the *quality* of specific neighborhood features, rather than their presence or absence, is a more salient element of neighborhood conditions for child mental health. Third, future research may wish to consider comorbidities, including adjustment for internalizing symptoms in models predicting externalizing behaviors. Fourth, future studies may wish to restrict analyses to urban areas to account for inherent differences in neighborhood context in rural versus urban settings. The current results are specific to children’s externalizing outcomes during middle childhood, and do not apply to adolescents or younger children. Future studies should consider the role developmental timing has in the effects of the neighborhood and by doing so, inform our understanding of contextual effects on child externalizing psychopathology. Finally, we examined the family’s current address in this study, and did not consider how long the family had been in residence at that address. Future studies should explore length of time in residence as a possible moderator of these associations.

## Conclusion

Several independent lines of research point to important associations between child mental health and their social, built, and toxicant neighborhood environments, respectively. Our study is the first to examine broader neighborhood exposome effects on child externalizing outcomes, and to illuminate how the various elements of the neighborhood exposome might jointly or synergistically influence child health outcomes. When evaluated separately, we found that built conditions were associated with conduct problems and toxicant conditions were associated with oppositional defiance problems. Further, the built environment was independently associated with child conduct problems when accounting for all neighborhood conditions. These results provide important information on *the specific elements of neighborhood* that are cross-sectionally related to children’s externalizing problems. Future studies should consider the role of development in these associations, both at the level of the child (who is growing each day) but also at the level of the neighborhood (since neighborhoods themselves change over time). For example, it could be that neighborhood associations with youth externalizing are strongest during adolescence in the presence of neighborhood decay over time. Such work would not only inform our understanding of contextual influences youth externalizing psychopathology, but would also illuminate the emergent qualities of neighborhoods.

## Supplementary Information

**Additional file 1: Table S1.** Child construct descriptive statistics before transformation. **Table S2.** Child construct descriptive statistics after transformation. **Table S3.** Environment indicator descriptive statistics before transformation within 5 km. **Table S4.** Environment indicator descriptive statistics after transformation within 5 km. **Table S5.** Environment indicator descriptive statistics before transformation within 1 km. **Table S6.** Environment indicator descriptive statistics after transformation within 1 km. **Table S7.** Correlations between informant reports of child oppositional defiance problems. **Table S8.** Correlations between informant reports of child conduct problems. **Table S9.** Correlations between environmental conditions. **Table S10.** Affective problems, anxiety problems, and ADHD problems association with the built condition. **Table S11.** Affective problems, anxiety problems, and ADHD problems association with the toxicant condition. **Table S12.** Affective problems, anxiety problems, and ADHD problems association with the toxicant condition. **Table S13.** Affective problems, anxiety problems, and ADHD problems association with the toxicant condition. **Table S14.** Oppositional defiance and conduct problems associations with all neighborhood conditions combined (except greenspace) with interactions. **Table S15.** Affective, anxiety, and ADHD problems associations with the built condition. **Table S16.** Oppositional Defiance and conduct problems association with built condition. **Table S17.** Affective, anxiety, and ADHD problems associations with the toxicant condition. **Table S18.** Oppositional Defiance and conduct problems association with toxicant condition. **Table S19.** Affective, anxiety, and ADHD problems associations with green space. **Table S20.** Oppositional Defiance and conduct problems association with green space. **Table S21.** Affective, anxiety, and ADHD problems associations with the social condition. **Table S22.** Oppositional Defiance and conduct problems association with the social condition. **Table S23.** Oppositional defiance and conduct problems associations with all neighborhood conditions combined (except greenspace). **Table S24.** PCA results for the built condition. **Table S25.** PCA results for the built condition without green space. **Table S26.** PCA results for the toxicant condition. **Table S27.** PCA results for the social condition. **Table S28.** PCA results for the built condition. **Table S29.** PCA results for the built condition without green space. **Table S30.** PCA results for the social condition. **Table S31.** CFA results for informants of oppositional defiance problems. **Table S32.** CFA results for informants of conduct problems.

## Data Availability

The datasets generated and/or analyzed during the current study are not publicly available due to the protection of the child participants’ home locations but are available from the corresponding author on reasonable request.
