# Supplementary material for: Associations between neighborhood built, social, or toxicant conditions and child externalizing behaviors in the Detroit metro area: a cross-sectional study of the neighborhood ‘exposome’
Source: BMC Public Health. 2022 May 28;22:1064. doi: 10.1186/s12889-022-13442-z (PMC9145391; doi:10.1186/s12889-022-13442-z)
Supplement: Supplementary file 1 — Additional file 1: Table S1. Child construct descriptive statistics before transformation. Table S2. Child construct descriptive statistics after transformation. Table S3. Environment indicator descriptive statistics before transformation within 5 km. Table S4. Environment indicator descriptive statistics after transformation within 5 km. Table S5. Environment indicator descriptive statistics before transformation within 1 km. Table S6. Environment indicator descriptive statistics after transformation within 1 km. Table S7. Correlations between informant reports of child oppositional defiance problems. Table S8. Correlations between informant reports of child conduct problems. Table S9. Correlations between environmental conditions. Table S10. Affective problems, anxiety problems, and ADHD problems association with the built condition. Table S11. Affective problems, anxiety problems, and ADHD problems association with the toxicant condition. Table S12. Affective problems, anxiety problems, and ADHD problems association with the toxicant condition. Table S13. Affective problems, anxiety problems, and ADHD problems association with the toxicant condition. Table S14. Oppositional defiance and conduct problems associations with all neighborhood conditions combined (except greenspace) with interactions. Table S15. Affective, anxiety, and ADHD problems associations with the built condition. Table S16. Oppositional Defiance and conduct problems association with built condition. Table S17. Affective, anxiety, and ADHD problems associations with the toxicant condition. Table S18. Oppositional Defiance and conduct problems association with toxicant condition. Table S19. Affective, anxiety, and ADHD problems associations with green space. Table S20. Oppositional Defiance and conduct problems association with green space. Table S21. Affective, anxiety, and ADHD problems associations with the social condition. Table S22. Oppositional Defiance and conduct problems association wit [file 12889_2022_13442_MOESM1_ESM.docx]

**Supplemental Materials**

**Additional Descriptive Information**

Table S1. Child construct descriptive statistics before transformation

| Construct | Mean | SD | Min | Max | Skewness | Kurtosis |
| --- | --- | --- | --- | --- | --- | --- |
| Oppositional Defiance Problems | 3.14 | 1.68 | 0 | 10.33 | .83 | .79 |
| Conduct Problems | 1.46 | 1.79 | 0 | 14.00 | 2.43 | 8.43 |

*Note.* These statistics are reported from the raw variables before transformation.

Table S2. Child construct descriptive statistics after transformation

| Construct | Mean | SD | Min | Max | Skewness | Kurtosis |
| --- | --- | --- | --- | --- | --- | --- |
| Conduct Problems | .71 | .58 | 0 | 2.71 | .69 | -.17 |

*Note.* These statistics are reported from the log transformed variables. Oppositional defiance problems was not included in this table as it did not need to be log transformed.

Table S3. Environment indicator descriptive statistics before transformation within 5km

| Construct | Mean | SD | Min | Max | Skewness | Kurtosis |
| --- | --- | --- | --- | --- | --- | --- |
| Number of Non-profits | 30.68 | 26.64 | 0 | 206 | 2.18 | 9.34 |
| Number of Churches | 4.85 | 4.09 | 0 | 19 | 1.14 | .79 |
| Sum Toxicants | 6099723.45 | 11224736.60 | .00 | 81000000 | 3.00 | 11.49 |
| Number of Alcohol Outlets | 236.12 | 146.56 | 1 | 628 | .10 | -.92 |
| Number of Pre1978 Structures | 31518.41 | 29370.56 | 571 | 124035 | 1.07 | .35 |
| Percent of Green Space Areas | 4.44 | 4.34 | 00 | 26.10 | 2.27 | 6.25 |
| Percent Industrial Areas | 4.77 | 3.37 | .00 | 17.88 | .93 | .74 |
| Percent Agricultural Areas | 6.08 | 13.94 | .00 | 87.28 | 2.85 | 8.21 |
| Percent vacant lots | 8.85 | 4.78 | .81 | 23.41 | .63 | -.14 |

*Note.* These statistics are reported from the raw variables before transformation.

Table S4. Environment indicator descriptive statistics after transformation within 5km

| Construct | Mean | SD | Min | Max | Skewness | Kurtosis |
| --- | --- | --- | --- | --- | --- | --- |
| Number of Non-profits | 3.05 | 1.02 | 0 | 5.33 | -.81 | .30 |
| Number of Churches | 1.51 | .75 | 0 | 3.00 | -.31 | -.49 |
| Sum Toxicants | 12.73 | 4.81 | 0 | 18.21 | -1.79 | 2.22 |
| Number of Pre1978 Structures | 9.78 | 1.22 | 6.35 | 11.73 | -.50 | -.67 |
| Percent of Green Space Areas | 1.49 | .69 | .00 | 3.30 | .13 | -.01 |
| Percent Agricultural Areas | .82 | 1.29 | .00 | 4.48 | 1.40 | .45 |
| Built Condition (PCA Component) | .00 | 1.00 | -2.84 | .70 | -1.40 | .45 |
| Toxicant Condition (PCA Component) | .00 | 1.00 | -1.05 | 3.15 | .1.07 | .36 |
| Social Condition (PCA Component) | .12 | .98 | -2.02 | 3.00 | .26 | -.53 |

*Note.* These statistics are reported from the raw variables before transformation. Alcohol outlets, percent industrial areas, and percent vacant lots were not include because they did not need to be transformed.

Table S5. Environment indicator descriptive statistics before transformation within 1km

| Construct | Mean | SD | Min | Max | Skewness | Kurtosis |
| --- | --- | --- | --- | --- | --- | --- |
| Number of Non-profits | 1.08 | 1.67 | 0 | 12 | 2.79 | 10.54 |
| Number of Churches | .21 | .48 | 0 | 2 | 2.25 | 4.36 |
| Sum Toxicants | 193190.40 | 1595860.57 | .00 | 19707263.00 | 11.15 | 127.10 |
| Number of Alcohol Outlets | 10.02 | 9.00 | 0 | 55 | .97 | 1.38 |
| Pre1978 Structures | 1535.56 | 1399.42 | 9 | 6290 | 1.15 | .99 |
| Percent of Green Space Areas | 5.22 | 9.811 | .00 | 59.86 | 3.11 | 10.72 |
| Percent Industrial Areas | 4.36 | 8.44 | .00 | 47.22 | 2.83 | 8.17 |
| Percent Agricultural Areas | 4.69 | 13.70 | .00 | 79.30 | 3.33 | 10.48 |
| Percent vacant lots | 11.40 | 11.12 | .00 | 65.28 | 1.69 | 3.15 |

*Note.* These statistics are reported from the raw variables before transformation.

Table S6. Environment indicator descriptive statistics after transformation within 1km

| Construct | Mean | SD | Min | Max | Skewness | Kurtosis |
| --- | --- | --- | --- | --- | --- | --- |
| Number of Non-profits | .53 | .60 | .00 | 2.56 | .88 | .03 |
| Number of Churches | .14 | .30 | .00 | 1.10 | 1.91 | 2.15 |
| Sum Toxicants | 2.09 | 4.51 | .00 | 16.80 | 1.84 | 1.67 |
| Percent of Green Space Areas | 1.07 | 1.15 | .00 | 4.11 | .73 | -.60 |
| Percent Industrial Areas | .93 | 1.11 | .00 | 3.88 | .94 | -.33 |
| Percent Agricultural Areas | .55 | 1.16 | .00 | 4.39 | 2.10 | 3.04 |
| Percent vacant lots | 2.14 | .90 | .00 | 4.19 | -.08 | -.71 |

*Note.* These statistics are reported from the raw variables before transformation. Alcohol outlets and pre1978 were not include because they did not need to be transformed.

Table S7. Correlations between informant reports of child oppositional defiance problems

|  | Mother Report | Father Report | Teacher Report | Child Report |
| --- | --- | --- | --- | --- |
| Mother Report | 1 |  |  |  |
| Father Report | .56^**^ | 1 |  |  |
| Teacher Report | .16^**^ | .08 | 1 |  |
| Child Report | .26^**^ | .17^**^ | .07 | 1 |

*Note*. Correlations among raw variables. *p<.05, **p<.01

Table S8. Correlations between informant reports of child conduct problems

|  | Mother Report | Father Report | Teacher Report | Child Report |
| --- | --- | --- | --- | --- |
| Mother Report | 1 |  |  |  |
| Father Report | .50^**^ | 1 |  |  |
| Teacher Report | .39^**^ | .33^**^ | 1 |  |
| Child Report | .34^**^ | .25^**^ | .16^.^ | 1 |

*Note*. Correlations among raw variables. *p<.05, **p<.01

Table S9. Correlations between environmental conditions

|  | Built Condition | Toxicant Condition | Social Condition |
| --- | --- | --- | --- |
| Built Condition | 1 | **.18**** | **-.27**** |
| Toxicant Condition | .55^**^ | 1 | **-.20**** |
| Social Condition | .27^**^ | .63^**^ | 1 |

*Note.* *p<.05, **p<.01; 5km below the diagonal and 1 km above the diagonal

**Additional Problems 5km Analyses**

Table S10. Affective problems, anxiety problems, and ADHD problems association with the built condition

| Affective Problems | | | | | | | Anxiety problems | | | | | | ADHD Problems | | | | | |
| --- | --- | --- | --- | --- | --- | --- | --- | --- | --- | --- | --- | --- | --- | --- | --- | --- | --- | --- |
|  | B | SE B | *β* | 95%CI | p | r^2^ | B | SE B | *β* | 95%CI | p | r^2^ | B | SE B | *β* | 95%CI | p | r^2^ |
|  |  |  |  |  |  | .03 |  |  |  |  |  | .02 |  |  |  |  |  | .10 |
| Ethnicity | .04 | .02 | .08 | (-.00, .17) | .063 |  | .02 | .02 | .05 | (-.03, .13) | .242 |  | .05 | .03 | .08 | (-.01, .17) | .079 |  |
| Sex | .04 | .02 | .08 | (.01, .16) | .037 |  | -.04 | .02 | -.09 | (-.16, -.01) | .032 |  | .14 | .02 | .23 | (.16, .31) | .000 |  |
| Age | .01 | .01 | .03 | (-.06, .11) | .563 |  | .01 | .01 | .04 | (-.06, .13) | .434 |  | -.06 | .02 | -.17 | (-.24, -.09) | .000 |  |
| Population | -.01 | .03 | -.01 | (-.12, .10) | .857 |  | .00 | .03 | .01 | (-.09, .11) | .906 |  | .04 | .04 | .05 | (-.05, .15) | .336 |  |
| Built | .05 | .02 | .12 | (.02,.22) | .018^*^ |  | .05 | .02 | .11 | (.01, .20) | .027 |  | .02 | .03 | .03 | (-.08, .13) | .617 |  |
| AIC = 10923.82; BIC = 11047.46 | | | | | | | AIC = 10875.15; BIC = 10998.79 | | | | | | AIC = 11242.39; BIC = 11366.03 | | | | | |

| Affective Problems | | | | | | | Anxiety problems | | | | | | ADHD Problems | | | | | |
| --- | --- | --- | --- | --- | --- | --- | --- | --- | --- | --- | --- | --- | --- | --- | --- | --- | --- | --- |
|  | B | SE B | *β* | 95%CI | p | r^2^ | B | SE B | *β* | 95%CI | p | r^2^ | B | SE B | *β* | 95%CI | p | r^2^ |
|  |  |  |  |  |  | .02 |  |  |  |  |  | .02 |  |  |  |  |  | .10 |
| Ethnicity | .04 | .02 | .07 | (-.01, .08) | .158 |  | .02 | .02 | .03 | (-.03, .06) | .500 |  | .05 | .03 | .08 | (-.02, .11) | .133 |  |
| Sex | .03 | .02 | .08 | (-.00, .07) | .054 |  | -.04 | .02 | -.09 | (-.07, -.01) | .022 |  | .13 | .02 | .23 | (.09, .18) | .000 |  |
| Age | .01 | .01 | .03 | (-.02, .03) | .589 |  | .01 | .01 | .03 | (-.02, .04) | .462 |  | -.07 | .02 | -.17 | (-.10, -.03) | .000 |  |
| Population | .01 | .03 | .02 | (-.05, .07) | .703 |  | .01 | .03 | .03 | (-.04, .07) | .609 |  | .04 | .04 | .05 | (-.04, .12) | .353 |  |
| Toxicant | .03 | .03 | .06 | (-.03, .08) | .352 |  | .03 | .02 | .07 | (-.02, .08) | .234 |  | .01 | .04 | .02 | (-.06, .08) | .729 |  |
| AIC = 10646.35; BIC = 10769.99 | | | | | | | AIC = 10595.53 BIC = 10719.17 | | | | | | AIC = 10958.70; BIC = 11082.34 | | | | | |

Table S11. Affective problems, anxiety problems, and ADHD problems association with the toxicant condition

Table S12. Affective problems, anxiety problems, and ADHD problems association with the toxicant condition

| Affective Problems | | | | | | | Anxiety problems | | | | | | ADHD Problems | | | | | |
| --- | --- | --- | --- | --- | --- | --- | --- | --- | --- | --- | --- | --- | --- | --- | --- | --- | --- | --- |
|  | B | SE B | *β* | 95%CI | p | r^2^ | B | SE B | *β* | 95%CI | p | r^2^ | B | SE B | *β* | 95%CI | p | r^2^ |
|  |  |  |  |  |  | .02 |  |  |  |  |  | .02 |  |  |  |  |  | .10 |
| Ethnicity | .05 | .02 | .09 | (.00, .18) | .043 |  | .03 | .02 | .06 | (-.01,.07) | .183 |  | .06 | .03 | .08 | (-.00, .11) | .072 |  |
| Sex | .04 | .02 | .08 | (.00, .16) | .042 |  | -.04 | .02 | -.09 | (-.07, -.00) | .031 |  | .14 | .02 | .24 | (.09, .18) | .000 |  |
| Age | .01 | .01 | .03 | (-.06, .12) | .543 |  | .01 | .01 | .04 | (-.02,.04) | .424 |  | -.06 | .02 | -.16 | (-.09, .-.03) | .000 |  |
| Population | .03 | .03 | .05 | (-.04, .14) | .281 |  | .03 | .02 | .06 | (-.02,.08) | .197 |  | .05 | .03 | .07 | (-.02, .11) | .147 |  |
| Green Space | .00 | .03 | .01 | (-.07, .09) | .876 |  | -.01 | .03 | -.01 | (-.06, .05) | .839 |  | .02 | .03 | .03 | (-.04, .09) | .476 |  |
| AIC = 10625.29; BIC = 10748.93 | | | | | | | AIC = 10574.78; BIC = 10698.42 | | | | | | AIC = 10935.96; BIC = 11059.60 | | | | | |

Table S13. Affective problems, anxiety problems, and ADHD problems association with the toxicant condition

| Affective Problems | | | | | | | Anxiety problems | | | | | | ADHD Problems | | | | | |
| --- | --- | --- | --- | --- | --- | --- | --- | --- | --- | --- | --- | --- | --- | --- | --- | --- | --- | --- |
|  | B | SE B | *β* | 95%CI | p | r^2^ | B | SE B | *β* | 95%CI | p | r^2^ | B | SE B | *β* | 95%CI | p | r^2^ |
|  |  |  |  |  |  | .02 |  |  |  |  |  | .02 |  |  |  |  |  | .10 |
| Ethnicity | .04 | .03 | .09 | (-.01, .18) | .087 |  | .03 | .02 | .06 | (-.04, .15) | .230 |  | .04 | .04 | .06 | (-.05, .16) | .281 |  |
| Sex | .04 | .02 | .08 | (.00, .16) | .042 |  | -.04 | .02 | -.09 | (-.17, -.01) | .030 |  | .13 | .02 | .23 | (.15, .31) | .000 |  |
| Age | .01 | .01 | .03 | (-.06, .12) | .545 |  | .01 | .01 | .04 | (-.05, .13) | .422 |  | -.06 | .02 | -.16 | (-.24, -.09) | .000 |  |
| Population | .03 | .03 | .05 | (-.05, .15) | .350 |  | .03 | .03 | .06 | (-.04, .16) | .245 |  | .03 | .03 | .05 | (-.05, .14) | .355 |  |
| Social | .01 | .03 | .01 | (-.10, .13) | .857 |  | .00 | .02 | .00 | (-.10, .11) | .952 |  | .04 | .03 | .06 | (-.05, .17) | .282 |  |
| AIC = 10527.46; BIC = 10651.10 | | | | | | | AIC = 10477.01; BIC = 10600.65 | | | | | | AIC = 10837.20; BIC = 10960.84 | | | | | |

Table S14.Oppositional defiance and conduct problems associations with all neighborhood conditions combined (except greenspace) with interactions

| Oppositional Defiance Problems | | | | | | | Conduct problems | | | | | |
| --- | --- | --- | --- | --- | --- | --- | --- | --- | --- | --- | --- | --- |
|  | B | SE B | β | 95% CI | p | r^2^ | B | SE B | β | 95% CI | p | r^2^ |
|  |  |  |  |  |  | .09 |  |  |  |  |  | .12 |
| Ethnicity | .11 | .10 | .06 | (-.04, .16) | .246 |  | .05 | .04 | .08 | (-.03, .19) | .151 |  |
| Sex | .22 | .07 | .13 | (.05, .21) | .001^*^ |  | .15 | .02 | .25 | (.17, .33) | .000* |  |
| Age | .06 | .05 | .06 | (-.03, .14) | .203 |  | -.04 | .02 | -.11 | (-.19, -.03) | .009* |  |
| Population | .08 | .11 | .04 | (-.07, .14) | .477 |  | -.02 | .04 | -.02 | (-.14, .10) | .719 |  |
| Built | .41 | .36 | .24 | (-.18, .66) | .261 |  | .16 | .13 | .28 | (-.16, .72) | .209 |  |
| Toxicant | -.08 | .29 | -.05 | (-.39, .29) | .782 |  | -.07 | .10 | -.11 | (-.45, .23) | .521 |  |
| Social | .08 | .11 | .05 | (-.08, .17) | .479 |  | -.01 | .04 | -.01 | (-.13, .11) | .887 |  |
| BuiltxToxicant | .19 | .38 | .10 | (-.27, .46) | .613 |  | .09 | .13 | .12 | (-.25, .50) | .526 |  |
| BuiltxSocial | .13 | .13 | .06 | (-.06, .19) | .319 |  | -.02 | .04 | -.03 | (-.14, .09) | .639 |  |
| ToxicantxSocial | .07 | .14 | .05 | (-.13, .23) | .589 |  | .05 | .04 | .10 | (-.04, .24) | .156 |  |
| AIC = 17373.87; BIC = 17726.47 | | | | | | | AIC = 15812.42; BIC = 16165.02 | | | | | |

* With Bonferroni correction, the adjusted p-value for significance is 0.025.

**Sensitivity Analyses within 1km**

Number of nonprofits, number of churches, sum of toxicants, percentage green space, industrial land use, agriculture land use, and vacant properties all evidenced considerable skew and were log transformed prior to PCA analyses.

Table S15. Affective, anxiety, and ADHD problems associations with the built condition

| Affective Problems | | | | | | | Anxiety problems | | | | | | ADHD Problems | | | | | |
| --- | --- | --- | --- | --- | --- | --- | --- | --- | --- | --- | --- | --- | --- | --- | --- | --- | --- | --- |
|  | B | SE B | *β* | 95% CI | p | r^2^ | B | SE B | *β* | 95% CI | p | r^2^ | B | SE B | *β* | 95% CI | p | r^2^ |
|  |  |  |  |  |  | .03 |  |  |  |  |  | .02 |  |  |  |  |  |  |
| Ethnicity | .04 | .02 | .08 | (-.00, .17) | .060 |  | .03 | .02 | .05 | (-.03, .13) | .228 |  | .05 | .03 | .08 | (-.01, .17) | .083 | .10 |
| Sex | .04 | .02 | .08 | (.01, .16) | .036 |  | -.04 | .02 | -.08 | (-.16, -.01) | .035 |  | .14 | .02 | .24 | (.16, .31) | .000* |  |
| Age | .01 | .01 | .03 | (-.06, .12) | .543 |  | .01 | .01 | .04 | (-.05, .13) | .420 |  | -.06 | .02 | -.17 | (-.24, -.09) | .000* |  |
| Population | .01 | .03 | .02 | (-.09, .12) | .758 |  | .02 | .03 | .03 | (-.07, .13) | .566 |  | .03 | .04 | .05 | (-.05, .15) | .337 |  |
| Built | -.04 | .02 | -.08 | (-.17, .01) | .099 |  | -.03 | .02 | -.07 | (-16, .02) | .136 |  | .02 | .03 | -.04 | (-.13, .06) | .477 |  |
| AIC = 10983.63; BIC = 11107.27 | | | | | | | AIC = 10934.03; BIC = 11057.67 | | | | | | AIC = 11297.76; BIC = 11421.40 | | | | | |

*Note*. With Bonferroni correction the adjusted p value for significance is .01. * indicates significant according to the adjust p value.

Table S16. Oppositional Defiance and conduct problems association with built condition

| Oppositional Defiance Problems | | | | | | | Conduct problems | | | | | |
| --- | --- | --- | --- | --- | --- | --- | --- | --- | --- | --- | --- | --- |
|  | B | SE B | β | 95% CI | p | r^2^ | B | SE B | β | 95% CI | p | r^2^ |
|  |  |  |  |  |  | .07 |  |  |  |  |  | .10 |
| Ethnicity | .23 | .09 | .12 | (.03, .21) | .011 |  | .07 | .03 | .10 | (.01, .19) | .027 |  |
| Sex | .26 | .07 | .15 | (.08, .23) | .000* |  | .15 | .02 | .26 | (.18, .34) | .000* |  |
| Age | .07 | .05 | .06 | (-.03, .15) | .168 |  | -.04 | .02 | -.10 | (-.18, -.02) | .012 |  |
| Population | .18 | .10 | .09 | (-.01, .18) | .072 |  | .00 | .04 | .01 | (-.09, .10) | .919 |  |
| Built | -.13 | .07 | -.08 | (-.16, .01) | .076 |  | -.05 | .03 | -.08 | (-.17, .00) | .055 |  |
| AIC = 12502.42; BIC = 12626.06 | | | | | | | AIC =10941.50; BIC = 11065.14 | | | | | |
|  | | | | | | |  | | | | | |

*Note*. With Bonferroni correction the adjusted p value for significance is .01. * indicates significant according to the adjust p value.

| Affective Problems | | | | | | | Anxiety problems | | | | | | ADHD Problems | | | | | |
| --- | --- | --- | --- | --- | --- | --- | --- | --- | --- | --- | --- | --- | --- | --- | --- | --- | --- | --- |
|  | B | SE B | *β* | 95% CI | p | r^2^ | B | SE B | *β* | 95% CI | p | r^2^ | B | SE B | *β* | 95% CI | p | r^2^ |
|  |  |  |  |  |  | .02 |  |  |  |  |  | .02 |  |  |  |  |  | .11 |
| Ethnicity | .04 | .02 | .08 | (-.01, .17) | .064 |  | .03 | .02 | .05 | (-.03, .14) | .207 |  | .04 | .03 | .07 | (-.02, .16) | .144 |  |
| Sex | .04 | .02 | .08 | (.00, .16) | .046 |  | -.04 | .02 | -.09 | (-.17 ,-.01) | .029 |  | .13 | .02 | .23 | (.15, .30) | .000* |  |
| Age | .01 | .01 | .03 | (-.06, .12) | .512 |  | .01 | .01 | .04 | (-.05, .13) | .413 |  | -.06 | .02 | -.16 | (-.24, -.08) | .000* |  |
| Population | .02 | .03 | .04 | (-.05, .13) | .405 |  | .03 | .03 | .06 | (-.04, .15) | .236 |  | .03 | .03 | .04 | (-.05, .13) | .367 |  |
| Toxicant | .02 | .02 | .05 | (-.04, .13) | .273 |  | .01 | .02 | .02 | (-.07, .10) | .735 |  | .06 | .02 | .10 | (.02, .18) | .017 |  |
| AIC = 11085.89; BIC = 11209.53 | | | | | | | AIC = 11036.75; BIC = 11160.39 | | | | | | AIC = 11391.56; BIC = 11515.20 | | | | | |

Table S17. Affective, anxiety, and ADHD problems associations with the toxicant condition

*Note*. With Bonferroni correction the adjusted p value for significance is .01. * indicates significant according to the adjust p value.

Table S18. Oppositional Defiance and conduct problems association with toxicant condition

| Oppositional Defiance Problems | | | | | | | Conduct problems | | | | | |
| --- | --- | --- | --- | --- | --- | --- | --- | --- | --- | --- | --- | --- |
|  | B | SE B | β | 95% CI | p | r^2^ | B | SE B | β | 95% CI | p | r^2^ |
|  |  |  |  |  |  | .07 |  |  |  |  |  | .10 |
| Ethnicity | .23 | .09 | .12 | (.03, .21) | .012 |  | .07 | .03 | .11 | (.02, .20) | .022 |  |
| Sex | .25 | .07 | .15 | (.07, .23) | .000* |  | .15 | .02 | .26 | (.18, .34) | .000* |  |
| Age | .07 | .05 | .06 | (-.02, .15) | .155 |  | -.04 | .02 | -.10 | (-.18, -.02) | .013 |  |
| Population | .22 | .09 | .11 | (.02, .20) | .014 |  | .03 | .03 | .04 | (-.05, .13) | .375 |  |
| Toxicant | .07 | .08 | .04 | (-.04, .13) | .338 |  | .00 | .03 | .00 | (-.09, .09) | .993 |  |
| AIC = 12963.28; BIC = 13086.92 | | | | | | | AIC =11403.59; BIC = 11527.23 | | | | | |

*Note*. With Bonferroni correction the adjusted p value for significance is .01. * indicates significant according to the adjust p value.

Table S19. Affective, anxiety, and ADHD problems associations with green space

| Affective Problems | | | | | | | Anxiety problems | | | | | | ADHD Problems | | | | | |
| --- | --- | --- | --- | --- | --- | --- | --- | --- | --- | --- | --- | --- | --- | --- | --- | --- | --- | --- |
|  | B | SE B | *β* | 95% CI | p | r^2^ | B | SE B | *β* | 95% CI | p | r^2^ | B | SE B | *β* | 95% CI | p | r^2^ |
|  |  |  |  |  |  | .02 |  |  |  |  |  | .02 |  |  |  |  |  | .10 |
| Ethnicity | .05 | .02 | .09 | (.00, .18) | .047 |  | .03 | .02 | .06 | (-.02, .14) | .155 |  | .06 | .03 | .08 | (-.01, .17) | .070 |  |
| Sex | .04 | .02 | .08 | (.00, .16) | .046 |  | -.04 | .02 | -.08 | (-.16, -.00) | .041 |  | .14 | .02 | .24 | (.16, .32) | .000* |  |
| Age | .01 | .01 | .03 | (-.06, .12) | .548 |  | .01 | .01 | .04 | (-.05, .13) | .413 |  | -.06 | .02 | -.16 | (-.24, -.09) | .000* |  |
| Population | .03 | .03 | .05 | (-.04,.14) | .293 |  | .03 | .02 | .06 | (-.03, .15) | .165 |  | .05 | .03 | .07 | (-.02, .15) | .151 |  |
| Green Space | .00 | .02 | .01 | (-.08, .09) | .862 |  | -.02 | .02 | -.04 | (-.13, .04) | .336 |  | -.01 | .02 | -.01 | (-.09, .07) | .826 |  |
| AIC = 11332.07; BIC = 11455.71 | | | | | | | AIC = 11280.37; BIC = 11404.01 | | | | | | AIC = 11643.35; BIC = 11766.99 | | | | | |

*Note*. With Bonferroni correction the adjusted p value for significance is .01. * indicates significant according to the adjust p value.

Table S20. Oppositional Defiance and conduct problems association with green space

| Oppositional Defiance Problems | | | | | | | Conduct problems | | | | | |
| --- | --- | --- | --- | --- | --- | --- | --- | --- | --- | --- | --- | --- |
|  | B | SE B | β | 95% CI | p | r^2^ | B | SE B | β | 95% CI | p | r^2^ |
|  |  |  |  |  |  | .07 |  |  |  |  |  | .10 |
| Ethnicity | .26 | .09 | .13 | (.04, .23) | .006* |  | .07 | .03 | .11 | (.02, .20) | .014 |  |
| Sex | .27 | .07 | .16 | (.08, .24) | .000* |  | .15 | .02 | .26 | (.19, .34) | .000* |  |
| Age | .07 | .05 | .06 | (-.03, .15) | .163 |  | -.04 | .02 | -.10 | (-.18, -.02) | .014 |  |
| Population | .26 | .09 | .13 | (.04, .21) | .005* |  | .03 | .03 | .05 | (-.04, .14) | .315 |  |
| Green Space | -.08 | .06 | -.06 | (-.14, .03) | .188 |  | -.02 | .02 | -.04 | (-.13, .05) | .384 |  |
| AIC = 13206.91; BIC = 13330.55 | | | | | | | AIC =11647.16; BIC = 11770.80 | | | | | |

*Note*. With Bonferroni correction the adjusted p value for significance is .01. * indicates significant according to the adjust p value.

Table S21. Affective, anxiety, and ADHD problems associations with the social condition

| Affective Problems | | | | | | | Anxiety problems | | | | | | ADHD Problems | | | | | |
| --- | --- | --- | --- | --- | --- | --- | --- | --- | --- | --- | --- | --- | --- | --- | --- | --- | --- | --- |
|  | B | SE B | *β* | 95% CI | p | r^2^ | B | SE B | *β* | 95% CI | p | r^2^ | B | SE B | *β* | 95% CI | p | r^2^ |
|  |  |  |  |  |  | .02 |  |  |  |  |  | .02 |  |  |  |  |  | .10 |
| Ethnicity | .05 | .02 | .09 | (-.01, .18) | .054 |  | .03 | .02 | .06 | (-.03, .14) | .187 |  | .06 | .03 | .09 | (-.01, .18) | .065 |  |
| Sex | .04 | .02 | .08 | (.00, .16) | .046 |  | -.04 | .02 | -.09 | (-.16, -.01) | .031 |  | .14 | .02 | .24 | (.16, .31) | .000* |  |
| Age | .01 | .01 | .03 | (-.06, .12) | .552 |  | .01 | .01 | .04 | (-.05, .13) | .416 |  | -.06 | .02 | -.16 | (-.24, -.09) | .000* |  |
| Population | .03 | .03 | .05 | (-.05, .14) | .318 |  | .03 | .03 | .06 | (-.03, .15) | .202 |  | .05 | .03 | .07 | (-.02, .16) | .133 |  |
| Social | -.01 | .03 | -.02 | (-.13, .09) | .760 |  | .00 | .03 | .01 | (-.11, .13) | .879 |  | .02 | .03 | .03 | (-.08, .15) | .585 |  |
| AIC = 10081.13; BIC = 10204.77 | | | | | | | AIC = 10030.70; BIC = 10154.34 | | | | | | AIC = 10392.12; BIC = 10515.76 | | | | | |

*Note*. With Bonferroni correction the adjusted p value for significance is .01. * indicates significant according to the adjust p value.

Table S22. Oppositional Defiance and conduct problems association with the social condition

| Oppositional Defiance Problems | | | | | | | Conduct problems | | | | | |
| --- | --- | --- | --- | --- | --- | --- | --- | --- | --- | --- | --- | --- |
|  | B | SE B | β | 95% CI | p |  | B | SE B | β | 95% CI | p |  |
|  |  |  |  |  |  |  |  |  |  |  |  |  |
| Ethnicity | .26 | .09 | .14 | (.05, .23) | .005* |  | .08 | .03 | .11 | (.02, .21) | .016 |  |
| Sex | .26 | .07 | .15 | (.08, .23) | .000* |  | .15 | .02 | .26 | (.18, .34) | .000* |  |
| Age | .07 | .05 | .06 | (-.02, .15) | .149 |  | -.04 | .02 | -.10 | (-.18, -.02) | .015 |  |
| Population | .27 | .09 | .13 | (.04, .22) | .005* |  | .03 | .03 | .05 | (-.04, .14) | .302 |  |
| Social | .12 | .10 | .07 | (-.05, .19) | .243 |  | .02 | .03 | .04 | (-.06, .14) | .423 |  |
| AIC = 11956.34; BIC = 12079.98 | | | | | | | AIC =10396.74; BIC = 10520.38 | | | | | |

*Note*. With Bonferroni correction the adjusted p value for significance is .01. * indicates significant according to the adjust p value.

Table S23. Oppositional defiance and conduct problems associations with all neighborhood conditions combined (except greenspace)

| Oppositional Defiance Problems | | | | | | | Conduct problems | | | | | |
| --- | --- | --- | --- | --- | --- | --- | --- | --- | --- | --- | --- | --- |
|  | B | SE B | β | 95% CI | p |  | B | SE B | β | 95% CI | p |  |
|  |  |  |  |  |  |  |  |  |  |  |  |  |
| Ethnicity | .24 | .09 | .13 | (.03, .22) | .010 |  | .07 | .03 | .11 | (.02, .20) | .023 |  |
| Sex | .26 | .07 | .15 | (.08, .23) | .000* |  | .15 | .02 | .26 | (.19, .34) | .000* |  |
| Age | .08 | .05 | .07 | (-.02, .15) | .120 |  | -.04 | .02 | -.10 | (-.18, .-.02) | .014 |  |
| Population | .19 | .10 | .09 | (-.01, .19) | .069 |  | .01 | .04 | .01 | (-.09, .11) | .802 |  |
| Built | -.15 | .07 | -.09 | (-.17, -.00) | .050 |  | -.05 | .03 | -.09 | (-.18, -.01) | .030 |  |
| Toxicant | .08 | .08 | .05 | (-.04, .13) | .319 |  | -.00 | .03 | -.00 | (-.09, .08) | .922 |  |
| Social | .16 | .10 | .09 | (-.03, .21) | .125 |  | .03 | .03 | .06 | (-.05, .16) | .302 |  |
| AIC = 15781.24; BIC = 15982.73 | | | | | | | AIC =14222.29; BIC = 14423.78 | | | | | |

*Note*. With Bonferroni correction the adjusted p value for significance is .01. * indicates significant according to the adjust p value.

**Principal Components Analysis (PCA) 5km**

Table S24**.** PCA results for the built condition

| Indicator | Component 1 | Component 2 |
| --- | --- | --- |
| Number of non-profits | .908 | .039 |
| Number of churches | .755 | -.133 |
| Number of alcohol outlets | .910 | -.046 |
| Percent area green space | -.007 | .964 |
| Percent area agricultural | -.850 | -.250 |
| Percent area vacant properties | -.472 | .211 |

*Note.* Based on the above results, it seemed that green space was creating the second component. Therefore, we decided to have green space be its own indicator.

Table S25. PCA results for the built condition without green space

| Indicator | Component 1 |
| --- | --- |
| Number of non-profits | .908 |
| Number of churches | .754 |
| Number of alcohol | .910 |
| Percent area agricultural | -.850 |
| Percent area vacant properties | -.472 |

Table S26. PCA results for the toxicant condition

| Indicator | Component 1 |
| --- | --- |
| Percent area industrial | .819 |
| Total Toxicants released | .858 |
| Number of pre 1978 structures | .560 |

Table S27. PCA results for the social condition

| Indicator | Component 1 |
| --- | --- |
| ADI State Level Disadvantage 2015 | .828 |
| Neighborhood Poverty level 2008- 2012 | .740 |
| Time 1 social cohesion | -.642 |
| Time 1 informal social control | -.818 |

**Principal Components Analysis (PCA) 1km**

Table S28**.** PCA results for the built condition

| Indicator | Component 1 | Component 2 |
| --- | --- | --- |
| Number of non-profits | .717 | -.274 |
| Number of churches | .494 | .028 |
| Number of alcohol | .754 | -.111 |
| Percent area green space | .419 | .647 |
| Percent area agricultural | -.676 | -.356 |
| Percent area vacant properties | -.397 | .619 |

*Note.* Based on the above results, it seemed that green space was creating the second component. Therefore, we decided to have green space be its own indicator.

Table S29. PCA results for the built condition without green space

| Indicator | Component 1 |
| --- | --- |
| Number of non-profits | .741 |
| Number of churches | .499 |
| Number of alcohol | .787 |
| Percent area agricultural | -.650 |
| Percent area vacant properties | -.419 |

Table S30. PCA results for the social condition

| Indicator | Component 1 |
| --- | --- |
| ADI State Level Disadvantage 2015 | .815 |
| Neighborhood Poverty level 2008- 2012 | .790 |
| Time 1 social cohesion | -.553 |
| Time 1 informal social control | -.733 |

**Confirmatory Factor Analyses (CFA) Results for Externalizing Informants**

To assess the underlying structure of the different information reports for oppositional defiance and conduct problems, we conducted one factor CFAs for each construct. The following variables were skewed and thus log transformed prior to analysis: Teacher report of oppositional defiance and conduct problems, mother report of conduct problems, dad report of conduct problems and child report of conduct problems. The below results suggest that the single factor model fits the data well.

Table S31. CFA results for informants of oppositional defiance problems

| Indicator | Factor loadings |
| --- | --- |
| Mother Report | .794 |
| Father Report | .731 |
| Teacher Report | .316 |
| Child Report | .310 |

*Note.* Standardized factor loadings are reported.

*χ^2^*(2) = 7.93, RMSEA = .06, CFI = .97, SRMR = .03.

Table S32. CFA results for informants of conduct problems

| Indicator | Factor loadings |
| --- | --- |
| Mother Report | .747 |
| Father Report | .653 |
| Teacher Report | .453 |
| Child Report | .411 |

*Note.* Standardized factor loadings are reported.

*χ^2^*(2) = .90, RMSEA = .00, CFI = 1.00, SRMR = .01.
